# Photovoice as a Method for Revealing Community Perceptions of the Built and Social Environment

**DOI:** 10.1177/160940691101000201

**Published:** 2011-01-01

**Authors:** Candace I.J. Nykiforuk, Helen Vallianatos, Laura M. Nieuwendyk

**Affiliations:** Centre for Health Promotion Studies, School of Public Health, University of Alberta, Edmonton, Canada; Department of Anthropology, University of Alberta, Edmonton, Canada; Centre for Health Promotion Studies, School of Public Health, University of Alberta, Edmonton, Canada

**Keywords:** photovoice, qualitative methods, community based participatory research, built environment, social environment

## Abstract

Over the last number of years there has been growing interest in the use of community-based participatory research (CBPR) for preventing and controlling complex public health problems. Photovoice is one of several qualitative methods utilized in CBPR, as it is a participatory method that has community participants use photography, and stories about their photographs, to identify and represent issues of importance to them. Over the past several years photovoice methodology has been frequently used to explore community health and social issues. One emerging opportunity for the utilization of photovoice methodology is research on community built and social environments, particularly when looking at the context of the neighbourhood. What is missing from the current body of photovoice literature is a critique of the strengths and weaknesses of photovoice as a method for health promotion research (which traditionally emphasizes capacity-building, community-based approaches) and as a method for revealing residents’ perceptions of community as a source of health opportunities or barriers. This paper will begin to address this gap by discussing the successes and challenges of using the photovoice methodology in a recent CBPR project to explore community perceptions of the built and social environment (with the ultimate goal of informing community-based chronic disease prevention initiatives). The paper concludes with methodological recommendations and directions for future research.

## Introduction

Community-based participatory research (CBPR) has become a popular approach for preventing and controlling complex public health problems (Horowitz, Robinson & Seifer, 2009; Israel, Schulz, Parker & Becker, 1998). This collaborative research approach employs community action and social change to effectively improve the health and well-being of the communities affected by the issue(s) under study (Israel, Schulz, Parker, & Becker, 2001; Minkler, Blackwell, Thompson & Tamir, 2003). CBPR integrates the research process with community capacity-building principles to bridge knowledge development and health promotion practice in communities; thus, it is well suited to upstream, or ecological, interventions that emphasize policy and environmental change (Israel et al., 2006). While the collaborative, capacity building intent of the CBPR approach has been clearly established, the methods for conducting this emerging body of work continue to evolve.

Photovoice (Wang & Burris, 1994, 1997) is one of several qualitative methods utilized in CBPR. It is a participatory method that has participants use photography, and stories about their photos to identify and represent issues of importance to them, which enables researchers to have a greater understanding of the issue under study (Nowell, Berkowitz, Deacon, & Foster-Fishman, 2006; Palibroda, Krieg, Murdock, & Havelock, 2009; Wang, 2006). Utilization of photovoice in conjunction with both community knowledge and best practice evidence can lead to the development of effective and comprehensive strategies to address complex health and social issues in a way that is also meaningful for the community involved.

## A Brief Overview of Photovoice and its Benefits and Challenges

The term photovoice was originally proposed by Wang and Burris in the early 1990s to describe the approach of blending narrative with photography to explore community issues; however this methodology builds on a deep, historical foundation of individuals and communities blending images and words to express needs, history, culture, problems, and desires (Collier & Collier, 1986; Pink, 2001; Pink, Keurti, & Afonso, 2004; Schwatz, 1989). The photovoice methods suggested by Wang and Burris (1994, 1997; Wang, 1999) included a number of distinct steps outlining participant and policy-maker recruitment and data collection. According to this approach, participants share photographs in a group setting through a facilitator-guided focus group discussion about (1) the key photographs selected by individuals in the group and (2) how to share information with policy-makers (for details, see Wang, 1999).

Photovoice has gained popularity as a qualitative research method that permits researchers from various disciplines to visualize individuals’ perceptions about their everyday realities (Close, 2007; Foster-Fishman, Nowell, Deacon, Nievar, & McCann, 2005). Photovoice projects have been conducted with a variety of cultures and population groups to explore a range of factors relating to health and social inquiry (Carlson, Engebretson, & Chamberlain, 2006; Castelden, Garvin, & Huu-ay-aht First Nation, 2008; Darbyshire, MacDougall, & Schiller, 2005; Downey, Ireson, & Scutchfield, 2009; Kofkin-Rudkin & Davis, 2007; Lockett, Willis, & Edwards, 2005; Mitchell & Kearns, 2007; Moffitt & Robinson-Vollman, 2004; Wang & Pies, 2004; Wang & Redwood-Jones, 2001; Wilson et al., 2007). The growing use of photovoice may be attributed to the numerous benefits it can provide for all stakeholders involved in the project (i.e., participants, researchers, the broader community, and decision-makers).

For participants, the photovoice process provides an opportunity to visually portray experiences and share personal knowledge about particular issues that may be difficult to express with words alone (Wang & Burris, 1997). This active engagement of community members in the research process demonstrates to participants that they are valuable members of the research team (Moffitt & Robinson-Vollman, 2004), and may contribute to a sense of community ownership through participation in a project that will help draw attention to important community issues (Wang & Burris, 1997). For researchers, the use of photographs helps to kindle dialogue amongst participants about their perceptions of the issues under discussion; further, different ideas may be obtained than those gathered solely from interviews or focus groups (Darbyshire, MacDougall, & Schiller, 2005). It is the combination of the narrative and visual depictions that enhances the ability of researchers to accurately capture the meaning of an issue from the participant’s point of view (Harrison, 2002; McIntyre, 2003; Nowell et al., 2006). The resulting photo stories become a potentially rich platform from which researchers can offer a nuanced understanding of community issues to the scientific community – an advance that can inform appropriate intervention or action on health and social problems.

There is also significant value the photovoice process for the partner communities. The flexibility of the photovoice collaborative process is well suited to CBPR projects, allowing it to be adopted in ways that can meet different communities’ needs. Further, the co-production of project results by the participants and researchers increases the saliency of findings for the community. Increased meaning in the results can be used to influence actions, policies and decisions impacting the community (Wang & Burris, 1997), thus enhancing the potential impact of projects at the local level. Finally, the photographs help provide a mechanism for decision-makers to see and understand residents’ perceptions of the health or social issue that needs to be addressed. Participants often identify innovative solutions to problems that would not normally be recognized by decision-makers, yet, the photo stories may elicit intuitive reactions from decision-makers that can foster action on community issues that accounts for their constituencies’ points of view (Wang & Burris, 1994).

While the benefits of photovoice have contributed to its increased use by qualitative researchers working in partnership with community groups, this methodology also presents challenges for the ethical and rigorous conduct of applied research. There is an inherent assumption with photovoice that its results will ultimately stimulate change by influencing decisions and policies at the community level. Yet, this can only occur if the project is backed by the political desire to change within the community. Lack of relationship building prior to beginning the photovoice process can create ethical dilemmas about raising awareness and expectation among participants and other community partners, while maintaining the status quo (i.e., no viable opportunity for community action or change (Wang & Burris, 1994; Wang & Redwood-Jones, 2001)).

The methodological challenges raised in the use of photovoice are further complicated when used as part of a community-based participatory method. The photographs and dialogue may be relatively straightforward to collect (once recruitment has occurred), but researchers still need to grapple with how to: (1) engage decision-makers to take part in the process; (2) sort and analyze the abundance of data resulting from data collection; and (3) ensure that the photographs and narratives are presented in a way that accurately portrays the participants’ most important issues (Wang & Burris, 1997; Wang & Redwood-Jones, 2001). The photographs themselves also present challenges: Evans (1999), for example, suggested that inaccuracy in results may be a consequence of participants’ subjective experiences being influenced by their personality or mood at the time of data collection. Yet, as Wachs (1999) stated, “what may be critical in determining individual behaviour patterns may be how the individual perceives the nature of his or her environment rather than the actual environment” (p. 366). Therefore, it is essential to capture how individual personality and mood influences perceptions, rather than images alone. Finally, while the photovoice process presents a distinctive way to understand individual perceptions of an issue, the process is very time consuming and requires a large investment of both human and financial resources.

## Plan of the Article

The obvious strengths of photovoice as a qualitative research methodology to explore community health and social issues have led to its increased use for CBPR projects. One emerging opportunity for the utilization of photovoice methodology is for research on community built and social environments, particularly when examining the neighbourhood context (Nicotera, 2007). Despite a few notable exceptions, there is currently a paucity of literature that specifically uses photovoice to understand perceptions of built or community environments. Reported studies have broadly explored the concept of community environment, for example, individuals’ connections with their neighbourhood or community (Kofkin-Rudkin & Davis, 2007), or the meanings associated with the most important characteristics in the community (Nowell et al., 2006).

What is missing from the current body of photovoice literature is a critique of the strengths and weaknesses of photovoice as a method for health promotion research (which traditionally emphasized capacity-building, community-based approaches) and as a method for revealing residents’ perceptions of community as a source of health opportunities or barriers. This paper will begin to address this gap by discussing the successes and challenges of using the photovoice method in a recent CBPR project to explore community perceptions of the built and social environment towards the ultimate goal of informing community-based chronic disease prevention initiatives. The built environment focus of the project emerged out of a community and research partnership interested in better understanding the relationships between health and place, specifically in the context of how community residents’ perceptions of place fosters, inhibits or prevents healthy lifestyle choices. Here, the use of photovoice facilitated extension of the traditional focus on the built (physical) characteristics of the community environment to include the social perspectives of place as well. Following a description of the current project context and the methodological framework, we discuss the strengths and weaknesses of using the photovoice methodology to understand individual and community perceptions of local built environments within a health promotion lens. The paper concludes with recommendations for future research.

## Background

This photovoice project was one phase of the larger Community Health and the Built Environment (CHBE) project underway in the province of Alberta, Canada. The purpose of CHBE was twofold: (1) to build a comprehensive understanding of the role of place in interventions for obesity reduction and chronic disease prevention; and (2) to facilitate identification of environmental factors that may foster, inhibit or prevent the implementation and success of community interventions aimed at improving health and well-being (Nykiforuk, et al., 2011a). The project was grounded in the principles of health promotion, which emphasize the importance of community engagement, social justice and capacity building in the collective strategies of healthy public policy, creating supportive environments, strengthening community action, developing personal skills, and reorienting health services (WHO, 1986). Specifically, the CHBE focus on investigating community (built and social) environment as a context for healthy eating and physical activity were framed according to the strategy of creating supportive environments. The community is a central setting for health promotion as it fosters simultaneous mobilization of grassroots and policy-level change to enhance health and recognizes the need for creating an equilibrium between top-down and bottom-up approaches (Mittlemark, 1999; Braunack-Mayer & Louise, 2008).

CHBE was a three-year project that occurred in four communities: the town of St. Paul; the town of Bonnyville; the city of Medicine Hat and its suburb, the town of Redcliff; and the community of North Central Edmonton. The partnership with these communities built on their previous involvement with another project undertaken by members of the research team (Raine, et al., 2010); this provided an opportunity to create synergy between initiatives and to ensure sustainability of initiatives beyond the term of projects. St. Paul and Bonnyville are two small municipalities located in northern Alberta, each with a population of about 5,000, and serving a larger population of 10,000 from surrounding communities. St. Paul has a rich agricultural tradition, while Bonnyville serves a strong oil and gas industry (Statistics Canada, 2006). North Central Edmonton is considered an urban inner-city community, comprised of 11 distinct, but contiguous, neighbourhoods (total population of 39,689) (City of Edmonton, 2005) within the urban core of the City of Edmonton, located in the northern half of the province. Medicine Hat and Redcliff are located in the southern-most part of Alberta (approximately 9 hours away Edmonton when travelling by car). Medicine Hat is a large urban municipality with a total population of 56,997 (Statistics Canada, 2006) and major sources of industry include agriculture, manufacturing, and oil and gas. Redcliff borders Medicine Hat and shares a large number of resources and services with the larger municipality.

Following health promotion and CBPR principles, the CHBE research team partnered with key community stakeholders (e.g., decision-makers, not-for-profit organizations, general public, local health units, etc.) to form Community Working Groups (CWGs) in each of the four communities. The CWGs further utilized CBPR principles to collaboratively identify issues of interest and to develop, implement and evaluate community-specific interventions. This collaborative approach, considered in conjunction with each community’s attributes, dynamics and geographic locations, presented a unique opportunity to employ photovoice methodology and to compare its utilization in different settings. This methodology played out differently in each community, giving the CHBE team opportunity to reflect on the relative strengths and weaknesses of the photovoice method in the context of exploring community perceptions of the built environment.

## Method

Given the CBPR emphasis of this photovoice project, the target population for participation in each community was determined in partnership with the CWGs. Extensive deliberation within each CWG led to the decision to focus on the general population within their respective community, rather than on a specific sub-group (e.g., youth, seniors, immigrants). Each CWG separately emphasized their need to build a foundational understanding of how the ‘general community’ viewed the community environment relative to health opportunities and barriers before exploring the perspectives of particular sub-populations or interest groups. Thus, the CWGs shaped the research question to be addressed by photovoice: *How do different elements within the built environment help or hinder individual and community efforts to be physically active and eat healthy food in their communities*.

Recruitment began three weeks prior to the planned timing of the initial interview with participants. A variety of mechanisms were used in an attempt to recruit the general population, while giving opportunities for minority or harder-to-reach populations to also participate. Methods included articles in the local newspapers, display of posters at key locations throughout the communities (identified by the CWGs), and e-mail fan-outs through local organization mailing lists. For some communities these modes proved to be successful, but in others a more purposive recruitment strategy needed to take place to ensure a sufficient sample size. This purposive strategy had CWG members personally promote the project by speaking with individuals from the community to identify people who would be interested in taking part in the project.

The goal of purposive sampling was to have eight to ten individuals representing the general population from each of the four communities take part in the project, representing a total sample size of 32 to 40 participants. Recruitment resulted in a total of 35 participants; ten from St. Paul, seven from Bonnyville, ten from North Central Edmonton and eight from Medicine Hat and Redcliff. Refer to Table 1 for a demographic profile of the participants.

The photovoice project was conducted in five distinct phases, occurring sequentially over a three-month period in the spring of 2009. The project methods were developed by reviewing the relevant photovoice literature and through discussions with the CWGs and the research team about what would be appropriate for each community. Previous photovoice methods have typically utilized focus groups to gather information from participants through facilitated discussions (Lockett et al., 2005; Nowell et al., 2006; Wang, 1999; Wang 2006; Wang & Burris, 1997). For this project, the research team and CWGs determined that it would be more appropriate to have individual one-on-one interviews with the participants to allow for a more in-depth exploration of both individual and community issues. The CWGs also led the sharing of project results with their communities; CWGs identified meaningful communication targets, helped the research team to develop appropriate (in content and format) reports for the communities, engaged media, and facilitated the sharing and use of project results in various decision-making meetings. Some of the photo stories have been shared in community venues such as local council meetings, public library exhibits, community arts festivals, and stories in local newspapers. Throughout the duration of the project all matters concerning potential issues and future directions were discussed with each of the CWGs.

Before discussing the strengths and challenges of this participatory methodology, a more detailed description of the five-phase process is provided. Research ethics clearance was obtained from the Health Research Ethics Board (Panel B), University of Alberta prior to the start of this project.

### Phase 1: Initial Interview

The initial semi-structured interview, which lasted approximately one hour, provided an opportunity for the interviewer (a trained graduate-level research assistant) to build rapport with each participant. This interview was used as an opportunity to understand the participant’s perceptions of the community and to gain an appreciation for the individual’s ideas about physical activity and healthy eating. Interviews were conducted at central location in each community that was quiet and private (e.g., local library or community centre). Prior to the interview, the interviewer reviewed the project information letter with the participant and obtained informed consent. Interviews were audio-recorded and an observer was present to record notes and take part in the discussion, if needed. After the interview, participants were provided with a digital camera to take photographs over a two-week period. Participants were shown how to operate the camera and were provided with a ‘photography mission.’ The photography mission was a loosely structured photo-topic that suggested participants take photos of places or things that they felt helped or hindered them from being physically active or eating healthy food in their community. Participants were encouraged to interpret the photography mission in whatever way made the most sense for them. If a participant was confused about what they were supposed to do for the project, a few very general suggestions were provided to the participant by the interviewer at the initial interview phase (Wang & Redwood-Jones, 2001).

### Phase 2: Taking the Photographs

Participants were given two weeks to take photographs around their community. A toll-free phone number was provided to participants to allow them to contact the project coordinator, at no cost to themselves, as needed throughout the project. This was particularly important given the significant geographic distance between the research team (located in Edmonton) and three of the communities. There was no defined minimum or maximum number of photographs that should be taken, but participants were advised that in the follow-up interview there would only be time to discuss a handful of photos. Across communities, participants took a range of 9 – 182 photographs of their community, averaging 40 to 50 photos per participant.

### Phase 3: Follow-up Interview

The follow-up interview occurred two weeks after, and in the same setting as, the initial interview. The second interview was semi-structured, lasted approximately 90 minutes, and guided participants to tell a story about a handful of their photographs. The interviewer began by having the participant select the photograph that was most meaningful for him/her to discuss. The interviewer then asked the participant a series of questions about the photo (e.g., why is this picture important to you?). When there was no more to be said, the interviewer would ask the participant to select another photograph that was meaningful to him/her. This continued until most of the interview time had elapsed or until the participant began to show signs of boredom or fatigue. Following the discussion of the photographs, the participant was asked a number of questions about their experiences with the project and how participation had impacted his/her perspectives of the community. Due to the substantial time commitment required from participants to take part in this project, they were provided with a $30 gift certificate to a local grocery store following the completion of the second interview. Participants were also provided with a hard copy of all of their photographs to take home with them.

### Phase 4: Summarizing the Participants’ Key Photographs

**Figure 1:**
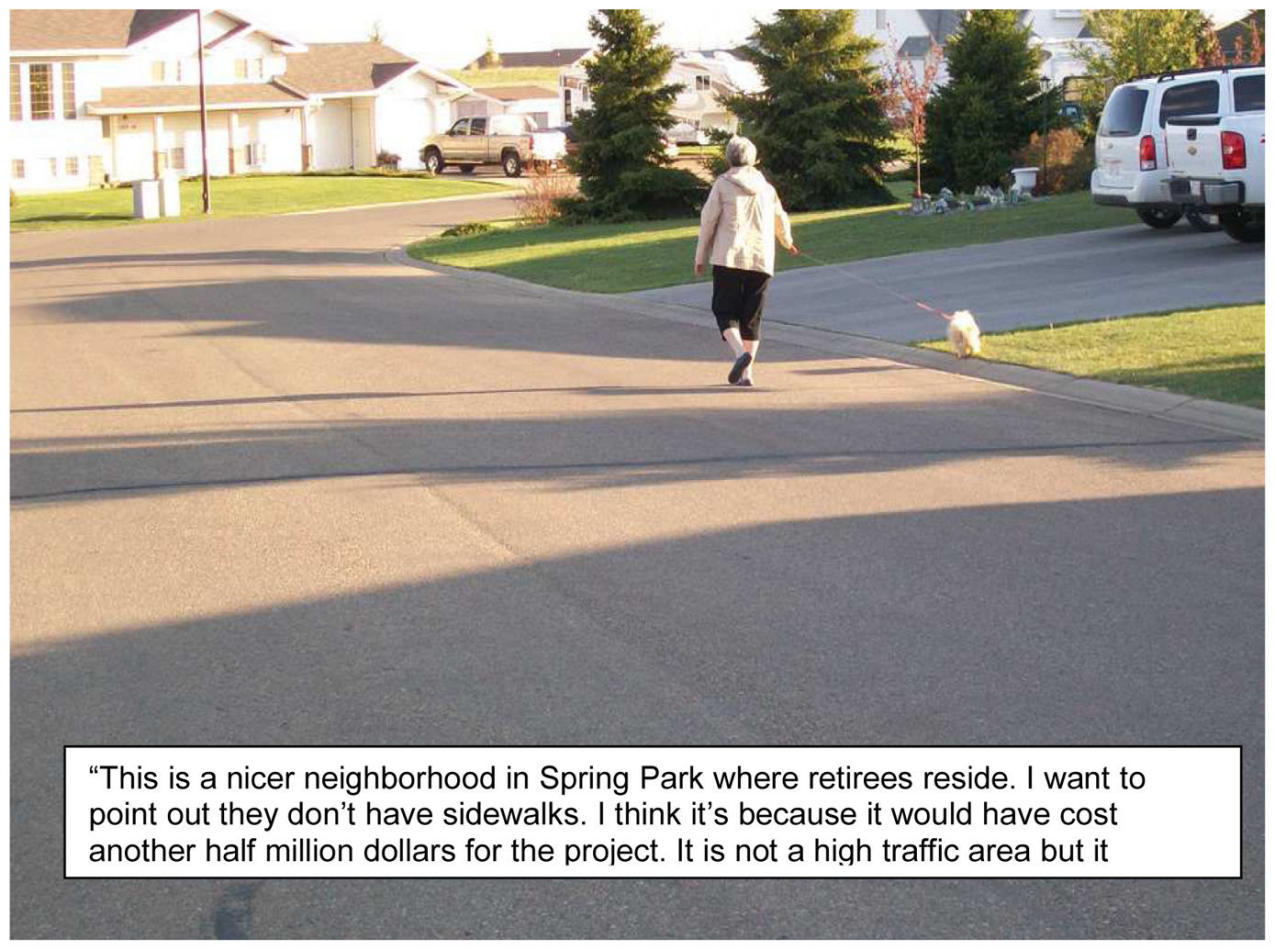
Example of a ‘Physical Activity’ Photo Story

**Figure 2:**
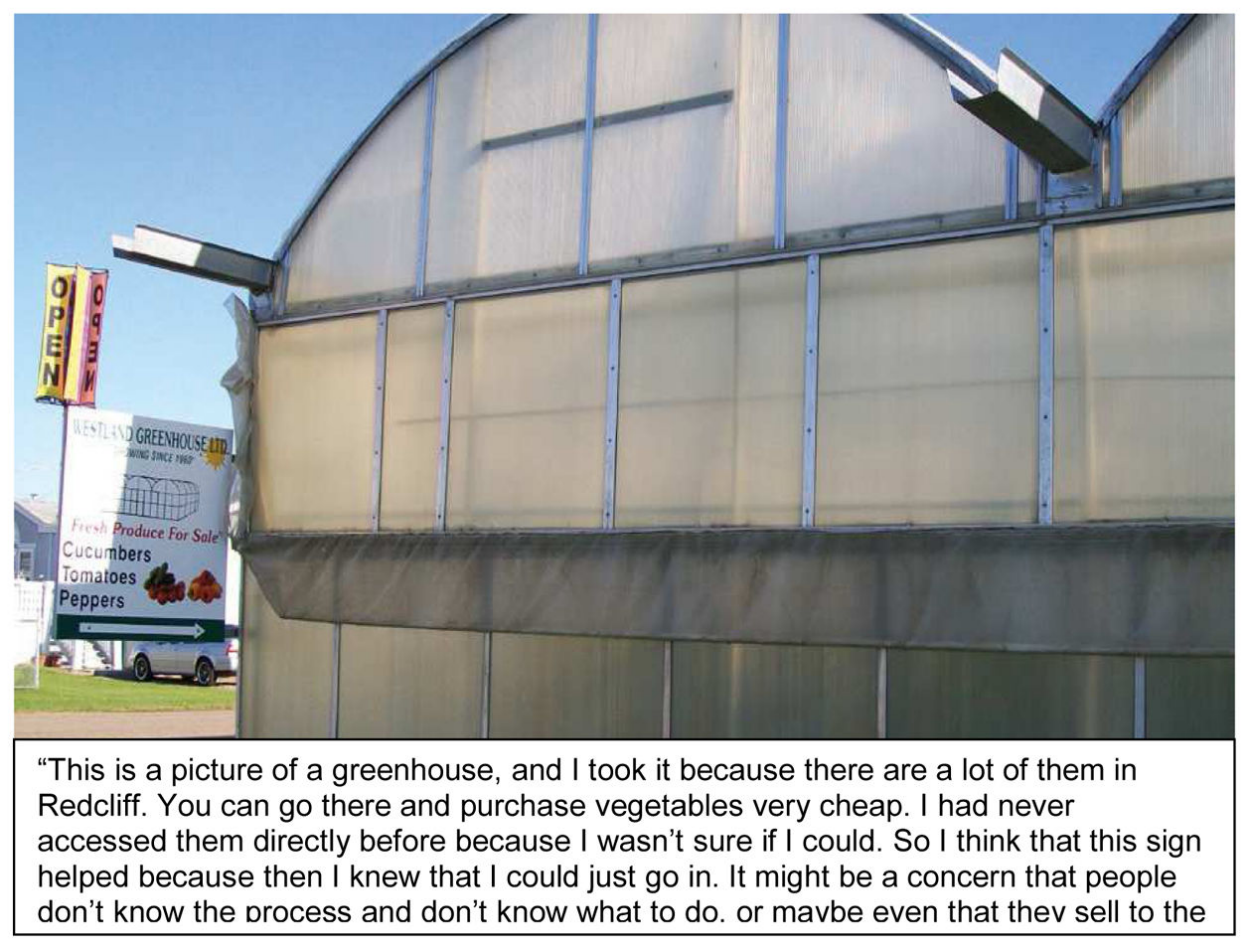
Example of a ‘Healthy Eating’ Photo Story

Following the interview, the top five photographs from those discussed by each participant were presented in a community presentation or display. The top five from each participant were selected as this allowed for the majority of each of the participants’ most meaningful photographs to be shared. A brief summary was written based on the interview transcripts to accompany each photograph. All of the photos and associated summaries were sent to the participant to review prior to being displayed in the community. This review process offered participants the opportunity to identify if they did not want a particular photograph displayed and to ensure that the written summary accurately reflected what they had intended it to reflect. All participants were successfully contacted and only a few participants (approximately five) had feedback about their summaries, particularly to do with wording choices; in each case, the participant’s revised wording was used. Participants were given the option of having their names associated with the photos or kept confidential (i.e., known only to a few members of the research team). Examples of participants’ photo stories are provided in Figures 1 and 2; analysis of photo stories beyond the preparation of summaries is detailed below figures.

### Phase 5: Presentation or Display of the Photographs

The presentation of the photo stories within each community was decided through discussions with the CWGs to determine the most appropriate venue and type of display. As co-owners of the photovoice data, it was decided that the research team would be responsible for publishing the project in scientific venues, while the CWG would be responsible for facilitating presentation of the stories in community venues. Because of the CWGs involvement and leadership in sharing the project results, the community photo stories were quickly (i.e., within 3 months of project completion) shared with the broader communities at local events (e.g., arts festivals and community events), presentations at local gathering places (e.g., community recreation centres and public libraries), on community websites, in local media stories, and through presentations to municipal councils.

## Analysis

In addition to the preparation of the summarized photo stories, this project resulted in a variety of data to be used for analysis including: participants’ photographs; verbal reflections on the photographs through the interview discourse; and background information about the participant collected at the initial interview phase. A thematic analysis was conducted to identify common codes and themes in the transcripts; results are reported elsewhere (Nykiforuk, et al., 2011b). Prior to analysis, a debriefing session was held with all interviewers and observers to ensure that the researchers were aware of the highs and lows from data collection; strengths and limitations of this process will be highlighted in the discussion section below. Using feedback from the debriefing session along with constructs identified through a comprehensive literature review and discussions with the CWGs, a list of codes was created by the research team to ensure that issues related to understanding perceptions of the community environment were identified during analysis. Three graduate-level research assistants went through an extensive training process to ensure that each individual was consistently coding the information. Inter-rater reliability was assessed through a systematic process of duplicate coding. All of the final interviews were coded by two of the research assistants and the initial interviews were coded by the third research assistant; 20% of the initial interviews were double-coded to check for inter-rater reliability. The project team met weekly during analysis to debrief about the coding process, discuss questions, and to identify any new emergent themes. Allowing space for inductive analysis is critical to the validity of qualitative research. Data which did not fit with one of the predetermined categories in the coding scheme was used to form new themes as appropriate. Following the initial coding, new themes were compared and collapsed into new categories where redundancy was identified. Coding memos were used to document all analytic decisions.

**Figure 3:**
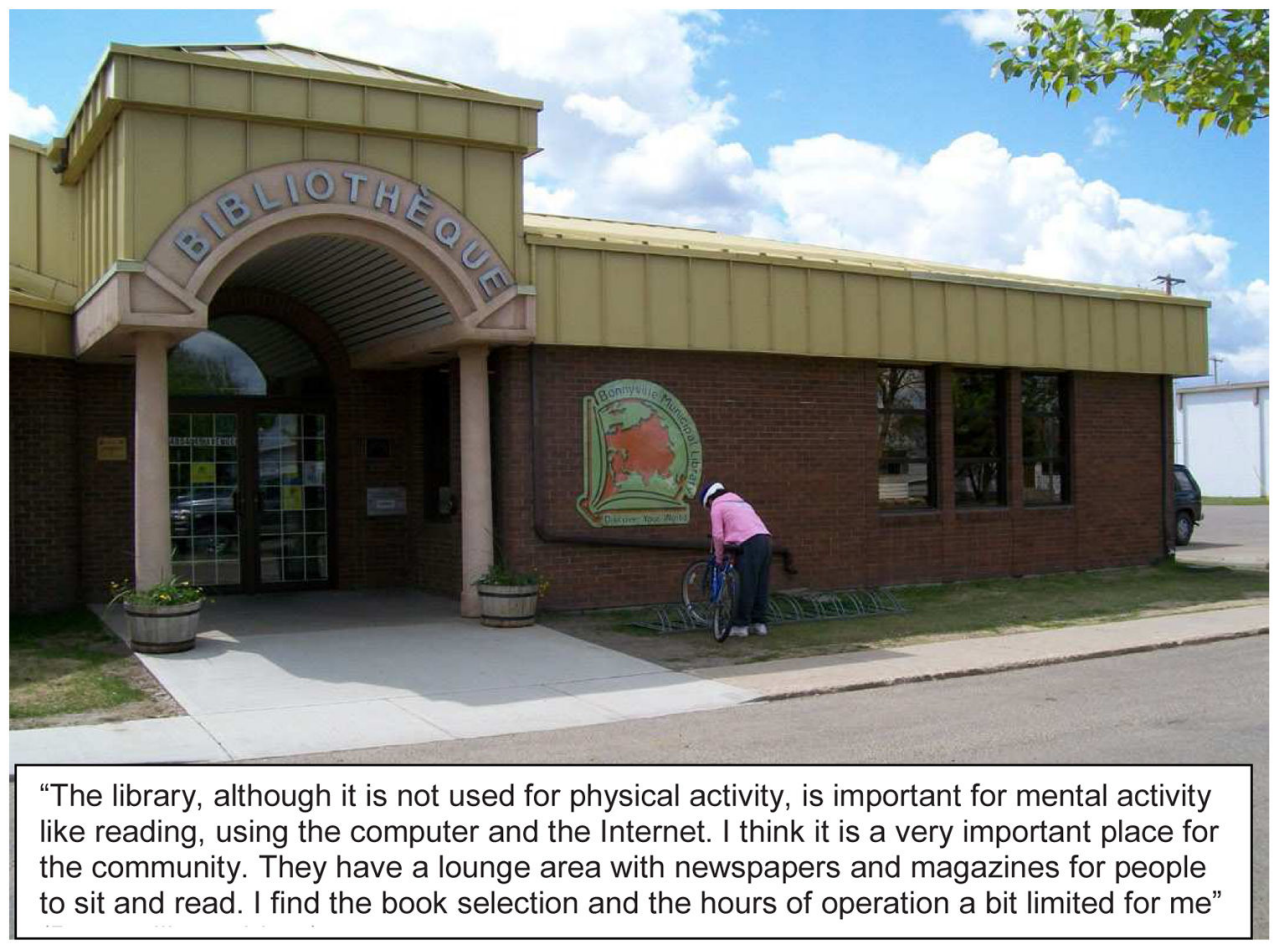
Example of a ‘Participant Issue - Libraries’ Photo Story

**Figure 4:**
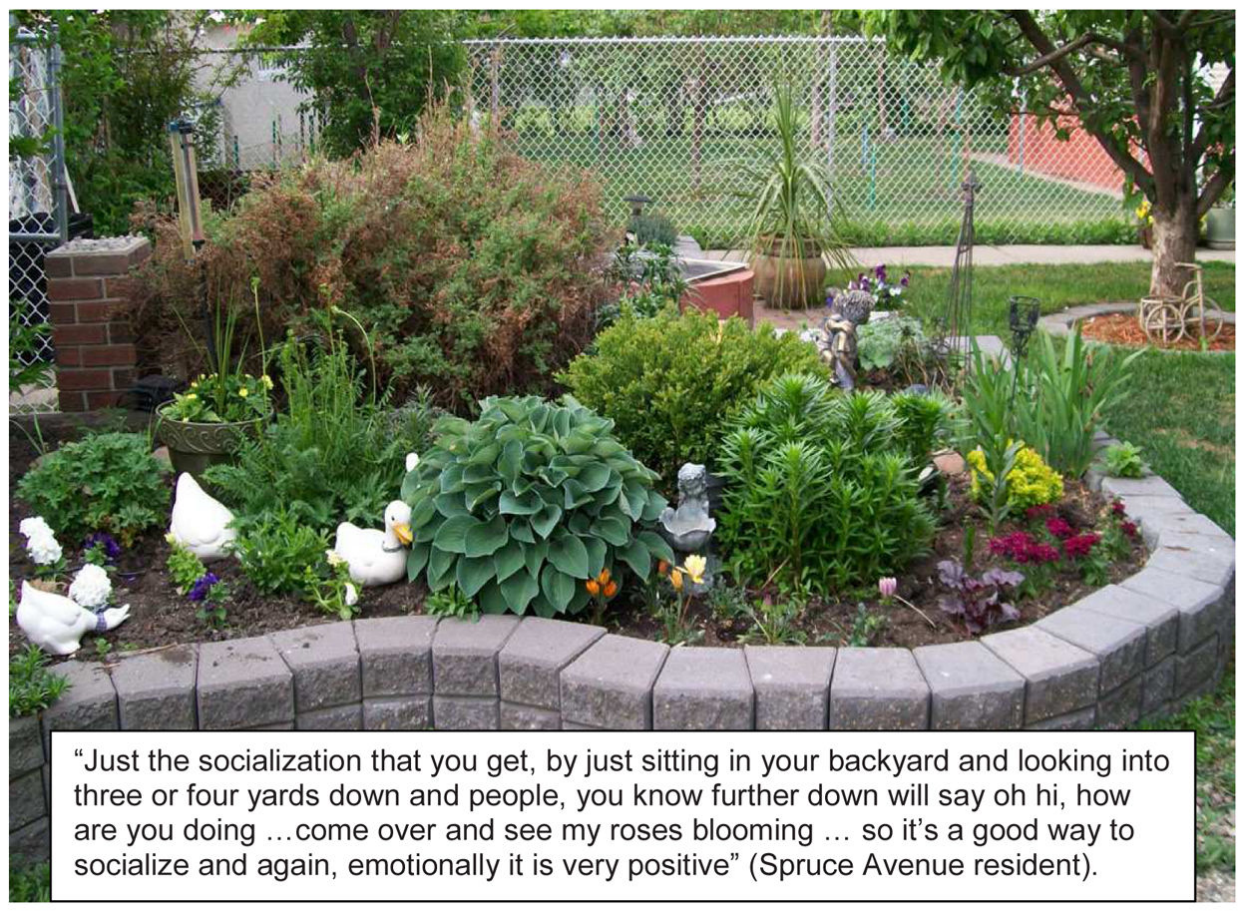
Example of a ‘Participant Issue - Community Connection’ Photo Story

The participants’ photographs were used to complement the thematic analysis of the interview data. It is important to highlight that since the interviews were focused on specific photographs from each participant, the analysis focused only on the dialogue associated with these photographs. In some cases, the photographs selected may not have represented the issues that the researchers and CWG had wanted to explore, but rather, were issues of importance to the participant; see Figures 3 and 4. While the participatory nature of this project rested primarily on the deep involvement of the CWGs as a cross-section of multi-sectoral community stakeholders, these participant-driven choices illustrate both the flexibility of the photovoice process (i.e., to be adaptive to circumstance) and the autonomy of participants to engage with the project in way that is significant for them.

The remaining photographs (i.e., those not discussed in the follow-up interview due to insufficient time or participant fatigue) were stored for potential future use by the communities. It was not appropriate to include these photographs in the analysis for research purposes because this would have involved the researchers, not the participants, ascribing meaning to the photos. Similar methods were used by Nowell and colleagues (2006) because there was acknowledgment that photographs used in the interview process were considered to be significant to participants, which had led to in-depth discussions about their significance, and contained the deepest and most critical discussions surrounding their content. In photovoice, it is not possible for researchers to authentically use the photographs for research purposes without the involvement of the participant.

## Discussion of Pitfalls and Lessons Learned

This project afforded our team the opportunity to critically reflect on the benefits and limitations of using photovoice methodology as highlighted by other researchers. Our intent herein was to provide a critique of how the photovoice methodology, used from a health promotion lens, contributed (or not) to gaining an understanding of residents’ perceptions of community environment relative to healthy eating and physical activity opportunities. Thus the question remains: did the photovoice method provide an appropriate means for community residents to portray their perceptions of their community environment? To answer this question it is necessary to understand the associated pitfalls, lessons learned, and the benefits of using the photovoice method in the current study. Our reflections on these issues have been delineated in a number of sub-sections to guide the reader through the discussion.

## Limitations related to Sampling

As noted, there was an initial series of meetings between the researchers and CWGs to select a target population for the project. The researchers anticipated that the communities would want to focus on a specific population group that aligned with some of the CBPR initiatives that were planned as part of the broader CHBE project. Such an approach would allow each of the communities to gather specific information about a particular population group and area of interest. However, the discussions with the CWGs led to each group independently choosing to focus the photovoice project on the general community population; this presented several challenges.

The limited sample size afforded by the photovoice method (in the context of the funding available for this project) did not permit recruitment of a large enough group of participants to ensure diversity through sample size alone. Therefore, despite the collective interest in ensuring a diverse sample, recruitment in each of the communities required a substantial level of coordination by the research team to ensure that the population diversity of each community was adequately, if nominally, represented. The greatest limitation of the CHBE photovoice project was the small sample size and gender imbalance in each community, despite concerted efforts by the research team and CWGs to achieve more balanced samples. Community samples ranged from seven to ten participants, 74.6% of whom were female (see Table 1). While these limitations could be framed as acceptable for the purposes of exploratory qualitative research, from a CBPR perspective, they hold serious implications for the use of the project results for community decision-making (i.e., because they are not representative of the general population of each community). While not possible to completely address this, when sharing the results with each community, the research team and CWGs fully disclosed these limitations and framed all findings and recommendations in light of the implications of such. The members of the CWGs, which included local decision-makers, also recognized the importance of (1) using other sources of community data to contextualize the photovoice findings for the purposes of decision-making, some of which emerged through discussions following community presentations, and (2) undertaking future projects that are tailored to gaining findings that are more generalizable to the community population.

As expected, data analysis has provided the CHBE team and CWGs with a broad understanding of residents’ perceptions rather than exposing issues of significance to specific target populations. While this specificity would be of value for the development of targeted interventions, the exploratory perspective provided by the results begins to tell a deeper story about the community environment as it relates to individual choices about health. For the research team, this was helpful with respect to informing subsequent CHBE intervention development that was consistent with developing personal skills and creating supportive environments. This bigger picture also was of great value to the community partners, and led to wholehearted buy-in (and requests) for future projects targeting particular sub-population groups or key locations. While the photovoice method necessitated a significant time investment to achieve the goals of both the community and research partners, it also created a strong foundation – and relationship – from which to continue collaborative work.

## Considerations of Geography and Self-Selection of Participants

Another challenge associated with the conduct of photovoice for understanding community environment is the geography of the communities under study. In the two semi-rural municipalities (Bonnyville and St. Paul) the populations and the geographic area were relatively small (i.e., populations of about 5000). In contrast, the two urban areas (North Central Edmonton and Medicine Hat/Redcliff and area) had much larger populations (i.e., 40,000 to 60,000 people) spread over large geographic areas. For the large urban areas, identification of key locations for recruitment was a significant challenge relative to that in the smaller communities. In contrast, the photographs and narratives from the urban areas represented a greater diversity of locations and issues than those from the concentrated semi-rural areas. For projects interested in revealing saturation rather than diversity, a focus on a small portion of the larger community or a specific neighbourhood would be advantageous over a whole-community lens.

A final challenge is related to the nature of participants who agree to participate in a photovoice project, and how well these individuals, who self-selected to take part in the project, represent their community. Participation in the current project required approximately five to ten hours of a participant’s time over a three-week period. Individuals that are willing to spend this amount of their personal time to participate in a community project tend to be those community members who are naturally more involved in the community, a bias which may limit the transferability of the findings to other settings (Nowell et al., 2006). Despite the limited transferability of the findings, findings from this type of research may still reveal an important association between places and people, and the transactions between them (Nowell et al., 2006).

These data collection challenges highlight a number of lessons and potential directions for future applications of the photovoice method. First, recruitment strategies should be multi-modal and take advantage of the expertise and credibility of the community partners. Word-of-mouth recruitment at community events and in informal settings, while labour-intensive, offered the best results for the time invested. Snowball sampling also presents an attractive opportunity to have recruited participants invite others to take part in the project.

## Insights on Methodology

Future photovoice projects considering the use of digital cameras are advised to set a maximum number of photographs to be taken for project purposes. In the current project, participants were provided with digital cameras with memory cards that held upwards of 500 photographs. Our team’s consultation with other researchers who had previously used photovoice found that participants typically did not take enough photographs; specifically, other researchers identified that they had never had a problem with participants taking too many photographs, therefore no limits were set. Thus, for this project, no formal limits were set on the number of pictures that could be taken, but following caution, it was suggested to participants that only a small number of photographs would be discussed during the follow-up interview. This method resulted in abundance of photographs for the current project; most participants took approximately 50 photos each, with a range of 9–182 photos taken across the 35 participants. This process resulted in an abundance of data that, along with the interviews, was relatively easy to collect, but created significant methodological challenges in terms of how the information should be analyzed and presented to ensure that participants’ perceptions were accurately portrayed (Wang & Burris, 1997).

Despite the large number of photographs taken by participants, asking them to select key photos for discussion during the follow-up interview worked very well. Approximately ten to twenty photographs were discussed per participant during the 90-minute follow-up interview. As 90 minutes was not enough time for the participant to discuss all of his/her photographs, there was a large number of leftover photographs that did not have the participant’s meaning attached to them (i.e., photos with no story, or photovoice without the voice (Darbyshire et al., 2005)). Yet, it remains inappropriate for researchers to ascribe meaning to these photographs. Therefore, it is necessary for researchers utilizing photovoice in the future to determine a way to ensure that, if in this situation, they discuss with participants if and how these additional photographs can be meaningfully integrated into the data analysis and presentation of community results. Despite the lack of symbolic value (meaning) of the leftover photos, the inherent value of the community images remains. In this case, permission was obtained from project participants for the leftover photos to be available for use by their community’s CWG for purposes related to the display of community images.

The project reported here employed a two-stage interview process involving an initial interview and single follow-up interview with each participant. This single follow-up interview with the participant provided only a snapshot of each person’s reality (Darbyshire et al., 2005). Future studies would benefit from the opportunity for additional follow-up either through multiple follow-up rounds of photo-taking or a series of interviews with participants to explore particular issues in greater depth (Rudkin & Davis, 2007).

The use of a photography mission in this project was intended to help focus the participant to take photographs of things that personally impacted their physical activity and healthy eating choices, yet the photographs that the majority of participants took were focused on the community. These photos reflected key destinations, locations and community assets that were available for the community to use. When participants were asked about their use of these facilities and destinations they suggested that they did not use them personally, but that these assets were available for the community to use. While this may indicate a mission vague in meaning, an alternative suggestion may be that there is an inherent disconnect between personal health choices and the array of food and physical activity resources in the community environment. At minimum, this disconnect emphasizes that the meanings of photos are dependent on what people have to say about them (e.g., if one were to just look at photos, one might interpret that the photographer is both knowledgeable of and an active user of the amenity, while, in reality, he/she may be knowledgeable, but not necessarily using the amenity). Further exploration of findings emerging from the analysis is discussed in a separate manuscript detailing the results of the CHBE photovoice project (Nykiforuk, et al., 2011b).

Many of these pitfalls and lessons learned resonate with an overarching methodological concern identified by Wang and Pies (2004), where the issues identified through photovoice are representative of a small sample of the population. Thus, if the process was to be repeated with a specific sub-sample of the population or with different people generally, the outcomes and results of the photovoice activity may be very different. Although this is an inherent limitation of photovoice, the method is, in fact, designed to allow people to represent their personal everyday realities. This presents an interesting question for the research community: is the photovoice method appropriate for broadly understanding residents’ perceptions (i.e., of the community (built and social) environment) or are the results only specific to the individual participant, and merely contextualized by the setting of the broader community?

## Reflections on the Participatory Nature of the Project

There are also methodological and CBPR-related issues associated with providing participants a photography mission. Such a method takes a step back from original photovoice methodology, where the focus of the photographs (and the project) is to be determined by the participants (Nowell, et al., 2006). The intensive engagement of the CWGs in the photovoice project (and in the overarching CHBE study) adhered to the participatory principles of CBPR, and involved the multi-sectoral CWG members in all aspects of the research process, i.e., from defining the research question, developing the coding and analytic frameworks, to sharing project results. However, at another level, the participatory nature of the photovoice project was limited in the extent to which individual participants were involved in specific elements of the research, i.e., the interpretation of the photography mission, creation of data through photography and narrative, selection of photos to be used for analysis, and review of photo stories.

The focus of the current project was collaboratively defined with each of the CWGs rather than with the individual participants. In order to preserve the opportunity for the participants’ voices to emerge, the research team and CWGs devised the guiding photography mission, rather than a ‘scavenger hunt’ list of specific statements (e.g., take pictures of places that you feel are attractive or unattractive). Thus, participants drove the mission by expressing their own interpretations through the photographs that they chose to take and speak to.

Subsequent to the follow-up interview, the research team selected the top five photographs from each participant’s interview to summarize for the community presentations. Again, this could be considered a limitation given that the photographs for the display were not re-selected by the participants themselves. This method was utilized to ensure that the photographs and accompanying stories represented issues that were relevant to the participant, the community, the researchers, and the funding body. While selecting the photographs for display, the research team took care to identify those photographs that the participant identified as being meaningful during the interview and subsequently reviewed the selection with the CWGs to ensure community relevancy.

Although this approach presented ethical dilemmas that have been encountered frequently in the use of photovoice, it is rarely identified as such in the literature (Moffitt & Robinson-Vollman, 2004; Wang & Redwood-Jones, 2001). To alleviate these ethical challenges, all of the selected photo stories were sent to the participants for re-review and consent for display, providing the participants with the final decision to include or withdraw a photograph and/or change the associated summary. As noted previously, the participants’ identification of the photographs that they wanted to discuss contributed to the participatory nature of the research, however, a limitation of this approach was that the selected photographs may not have represented the issues that the researchers and CWGs had wanted to explore and understand – requiring the project to negotiate and balance the various needs represented by the interests of the different project stakeholders. Further, flexibility in the exploratory stance was required in order to recognize that an individual’s personal judgments may interfere with the objective understanding of an issue (Wang & Burris, 1997). Adaptability in the selection of results to be shared with decision-makers in this case was illustrated by the resulting compromise between the levels of participation by the community as represented by the CWGs and the individual participants. This is consistent with previous work by Wang and colleagues that recommended research partners explore the degree of political desire to change, among other important community constructs, by engaging in dialogue and building an integral, communicative relationship with the community partners (Wang & Burris, 1994; Wang & Redwood-Jones, 2001).

The CHBE photovoice project attempted to strike a balance between the participation needs of the individual participants (bottom-up) and the community stakeholder members of the CWGs (top-down) in order to address the community issue (Braunack-Mayer & Louise, 2008). While the full potential of participation by individuals (photovoice participants) was not realized by this project, the extensive collaboration with CWGs and extent of engagement (and re-engagement) of participants was an authentic attempt to minimize tokenism. Still, this situation raises the critical question of whether or not the CHBE photovoice project was truly participatory. The field of health promotion traditionally rests on operationalization of the social ecological model, which recognizes that effective health promotion strategies must influence multiple levels, from the individual to community to public policy (McLeroy, Bibeau, Steckler, & Glanz, 1988). Yet, in the context of CBPR, there is little guidance offered by social ecological theory on how to effectively actualize participatory approaches across these multiple levels. CHBE, a health promotion community-research partnership that was intended to be participatory, was faced with the significant challenge of defining multi-level participation in its aim to meet community needs. The decision was made to move forward with the deep immersion of the CWGs and the preservation of autonomy for individual participants to engage in the project in a way that resonated with their personal values.

While many challenges were encountered, this approach was successful at multiple levels. At the individual level, the project worked well and resulted in tremendous buy-in and interest from participants, which raised their awareness concerning their own interactions with their community environments and led to their involvement in other community events and municipal council meetings. Sharing of project results (Phase 5) also promoted community action. For example, in one community, a presentation to municipal council resulted in immediate remediation of a community infrastructure problem identified through the project and consideration of CHBE data (including that from the photovoice project) in municipal planning documents. Despite this apparent success, two underlying question persist: was the project truly participatory?; and, is photovoice alone an appropriate methodology for meeting the demands of community action through citizen participation and decision-making in diverse communities? These authors invite reflection from the broader qualitative and CBPR research communities on these complex issues.

## Methodological Benefits

The many benefits realized from the use of photovoice in the current project highlight future directions for research despite the pitfalls encountered along the way. The photo stories provided an effective means for the CHBE researchers and CWGs to build an in-depth understanding of community issues from the residents’ perspectives. The decision to replace the focus group methods originally proposed by Wang and Burris (1994, 1997) with individual interviews was successful in the context of this project, and resulted in a rich qualitative data set. This decision also addressed the CWGs’ concerns about (1) the feasibility of getting enough participants to conduct multiple focus groups in the community and (2) people’s willingness to be open about their true perceptions in a group setting. The individual interviews provided a way to gather more in-depth information in a safe one-on-one setting.

Rapport, nature of dialogue, and the extent of issues discussed by participants were enhanced by the use of the two-stage interview method. Findings from the interviews suggested that the initial step revealed valuable information about the participants’ perspectives on the community in general prior to being assigned the photography mission. This information complemented the data collected through the follow-up interviews focused on the participant’s photos. Interviews proved to be a valuable mode for photovoice data collection, as this method allowed each participant to open up about their personal perceptions of the community. It is unlikely that the either the interviews or the photographs independently would have led to such rich understanding of the community. For example, the stories that accompanied the photographs allowed the participant to tell the researchers what was really going on in the photo and in the community. The photographs served as a catalyst to provide a view into the perspectives of the community from the eyes of those that live there. Without the visual imagery this would not have been evident (Rudkin & Davis, 2007).

While the photographs present a unique way for participants to express themselves, the photovoice process itself can be very beneficial for the participants. Engagement is inherent to the approach, giving community members the opportunity to have a voice through participation and visual representation of their community. Cameras are an appealing tool for the participants to use; the nature of photography lends itself to the active involvement of community members in a research process, which may increase participants’ motivation to improve their community (Rudkin & Davis, 2007). In the current project, participants noted that they learned about their community while participating in the project. For example, one participant stated, “This project made me look at myself and made me look at how I could fit better into my community,” while another commented that the project “certainly made me look around a lot more… now I am looking and trying to think, is that a street that I would want to walk on, what would draw me there, what keeps me away.” Thus, the participatory processes of photography and discussion in interviews (or focus groups) engages participants to re-examine their environments and place therein, leading to consciousness-raising. Through the process of taking photos, participants intuitively – and explicitly – reconsider issues that may lead to change in self and foster an impetus to participate in activities that stimulate action or create social change at the community level.

In addition to the personal learning opportunity afforded to the participants through the project, their photos and narratives are intended to contribute to local decision-making in support of physical activity and healthy eating (i.e., the participants are contributing to community change). Community and policy change are, however, slow processes; thus, the photovoice project must be implemented in a way that ensures there are opportunities for participants to benefit from the project beyond the distal potential to ‘influence change in your community.’ The photovoice process can help to provide an avenue for identifying community-driven interventions (Rudkin & Davis, 2007). This was especially important for CHBE given its goal to work collaboratively with the communities to develop, implement and evaluate community-driven interventions related to physical activity and healthy eating. The results of the photovoice project were particularly valuable for driving the overarching CHBE interventions, but also for influencing community-level decisions (Nykiforuk, et al., 2011a). The presentation of images and narratives of the various strengths and challenges associated with the built and social environment of each community have begun to impact decisions made within the community because (1) decision-makers were active participants in the CWGs and (2) all of the information presented was from the local context and expressed by local residents. This is a particular strength of the CHBE project, which recognized that researchers often engage communities in research, but rarely have the opportunity to present immediate, community-specific information back to decision-makers (Kelly, 2005; Rudd & Cummings, 1994).

Other researchers have noted ethical challenges with the use of photovoice because it assumes that the community-identified issues will be presented to decision-makers and policy-makers and that, subsequently, changes will occur (Wang & Redwood-Jones, 2001). The CHBE project addressed this through the initiation of the CWGs; these partnerships with local stakeholders facilitated access to many key decision-makers in the community and involved them in the project from its earliest stages. For example, the decisions about when and how to present the photo stories (e.g., electronically on a website or audio-visual presentation or through static displays) as well as the most appropriate locations for these presentations, were determined in partnership with the CWGs. Appropriate (and meaningful) collaboration with stakeholders and decision-makers in the conduct of a photovoice project can facilitate the use of photographic images to help drive the implementation of healthy public policy that addresses a community’s needs (Wang, 1999), and in the case of the current project, contribute to the creation of safe and health-enabling environments.

The myriad of benefits identified above demonstrate why, in the current study, use of photovoice was beneficial for understanding residents’ perceptions of their community’s built and social environment. While the interviews identified participants’ personal perceptions of access (or not) to health promoting opportunities as well as specific community-level opportunities and barriers to access, overall, the resulting photo stories helped to paint a picture of the community from ‘insider’ eyes. These results helped to provide a snapshot of the community that opened doors for decision-makers and the research team to explore and identify community-specific issues, strengths and gaps for future action. In the context of the CHBE photovoice project, the pictures were worth a thousand words when portraying local health promotion issues to decision-makers.

## Methodological Recommendations for Future Research

Several methodological recommendations arose through the conduct of the work reported here. First, it is strongly recommended that future photovoice projects employing digital cameras put a cap on the number of photographs that each participant can take as part of the project. If a cap is undesirable, sufficient time should be allocated during the interview (or focus group) session for discussion of all of the participant’s photographs. This would allow the researchers to gain in-depth information or the ‘whole story’ about the participant’s experience taking photos, rather than asking him/her to focus on a handful that are “the most meaningful or important to them.” Further, if the one-on-one interview method is followed, it is recommended that the project results be presented back to the community (and participants) in a focus group session to elicit further community feedback prior to broader community dissemination. This would help to assess to what extent the individual feedback collected from the participants resonates with the wider community perspective. This is particularly important given that the time required of the photovoice method necessitates a relatively small participant group.

Other methodological directions for future research include: accompanying participants while they are taking the photographs in ‘go-along’ photovoice methodology (Carpiano, 2009); use of videography where participants take live recordings of their experiences as they move through their ‘mission’ or explore their own perceptions of the stated project purpose (Fujita & Arikawa, 2008); or videographic go-alongs that combine the benefits of both alternative directions. These variations in methodology present different, and possibly more immediate, explorative opportunities for researchers and their community partners to understand why participants choose to capture images of some things and not others, and how they negotiate these decisions throughout the course of photo- or video-taking. This evolution of methods may provide even more nuanced information than traditional photovoice, thus permitting a more detailed story about the community or issue of interest.

## Conclusions

This health promotion photovoice project provided a rich opportunity to apply and critique the utility of the photovoice methodology for exploring residents’ perceptions of community across four different settings. Our adaptation of this methodology to a health promotion, built environment research question revealed that photovoice is an appropriate and compelling tool for this field, which is traditionally informed by quantitative approaches. Adoption of CBPR principles in the conduct of this photovoice project allowed for different permutations of the project to evolve in each of the four communities while maintaining the overall intent of the project. Despite slight variations in project implementation, broad similarities and differences in the themes emerging from photos and interviews were consistent across communities. Further, community partners (participants and CWG members) were unreservedly engaged in the projects, and began spearheading the sharing of results with their communities immediately upon release of the photo stories. This rapid knowledge exchange is particularly exciting for the emerging area of research on community environments, where capturing and communication distinctions between objective and subjective (perceived) environments, is critical for the development of ecological and community-based interventions to facilitate optimal access to health and social wellness.

## Figures and Tables

**Table 1:** Demographic of Participants in CHBE Photovoice Project

	*Bonnyville (n=7)*	*Medicine Hat and Redcliff (n=8)*	*North Central Edmonton (n=10)*	*St. Paul (n=10)*	*Total*
**Gender**					
Male	1 (14.3%)	3 (37.5%)	2 (20.0%)	3 (30.0%)	9 (25.7%)
Female	6 (85.7%)	5 (62.5%)	8 (80.0%)	7 (70.0%)	26 (74.3%)

**Age**					
Under 18	1 (14.3%)	1 (12.5%)	0	1 (10.0%)	3 (8.6%)
18–24	0	0	0	1 (10.0%)	1 (2.9%)
25–34	1 (14.3%)	3 (37.5%)	2 (20.0%)	0	6 (17.1%)
35–44	1 (14.3%)	1 (12.5%)	4 (40.0%)	4 (40.0%)	10 (28.6%)
45–64	2 (28.6%)	2 (25.0%)	2 (20.0%)	3 (30.0%)	9 (25.7%)
65+	2 (28.6%)	1 (12.5%)	2 (20.0%)	1 (10.0%)	6 (17.1%)

**Household Income**					
< $25,000/year	4 (57.1%)	1 (12.5%)	3 (30.0%)	4 (40.0%)	12 (34.3%)
$25,000 – $50,000/year	0	2 (25.0%)	0	0	2 (5.7%)
$50,000 – $75,000/year	2 (28.6%)	0	1 (10.0%)	1 (10.0%)	4 (11.4%)
$75,000 – $100,000/year	0	2 (25.0%)	4 (40.0%)	1 (10.0%)	7 (20.0%)
>$100,000/year	0	3 (37.5%)	1 (10.0%)	2 (20.0%)	6 (17.1%)
Prefer not to Answer	1 (14.3%)	0	1 (10.0%)	2 (20.0%)	4 (11.4%)
